# A Multilevel Intervention to Increase HPV Vaccination among Asian American Adolescents

**DOI:** 10.1007/s10900-021-01013-z

**Published:** 2021-07-07

**Authors:** Grace X. Ma, Lin Zhu, Yin Tan, Shumenghui Zhai, Timmy R. Lin, Cristina Zambrano, Philip Siu, Sarah Lai, Min Qi Wang

**Affiliations:** 1 Center for Asian Health, Lewis Katz School of Medicine, Temple University, 3440 N. Broad Street, Philadelphia, PA 19140 USA; 2 Department of Clinical Sciences, Lewis Katz School of Medicine, Temple University, 3440 N. Broad Street, Philadelphia, PA 19140 USA; 3 Fox Chase Cancer Center, Temple University Health System, 333 Cottman Ave, Philadelphia, PA 19111 USA; 4 Hunter College Center for Cancer Health Disparities Research, 695 Park Avenue, New York, NY 10065 USA; 5 Greater Philadelphia Health Action, Inc., 432 N 6th St, Philadelphia, PA 19123 USA; 6 School of Public Health, University of Maryland, 4200 Valley Dr, College Park, MD 20742 USA

**Keywords:** Human papillomavirus, Vaccination, Chinese American adolescents, Community-based participatory research

## Abstract

Human papillomavirus (HPV) is the most common sexually transmitted infection in the US. HPV vaccine is a viable source of prevention against high-risk strains that are likely to cause cancer. However, particularly among racial and ethnic minorities such as Chinese Americans, HPV vaccination rates are suboptimal. The goal of this study was to evaluate the effect of a culturally tailored intervention on HPV vaccine uptake in Chinese Americans. We designed and implemented a multilevel longitudinal pilot study to examine the efficacy of the HPV intervention among Chinese American parents/guardians. We recruited 180 participants from federally qualified health center and community-based clinics that serve predominantly low-income Chinese Americans in Philadelphia. Participants were randomized into an intervention group (n = 110) or a control group (n = 70). The intervention group received an HPV specific intervention, while the control group received a general health intervention. The primary outcome was medical record-confirmed receipt of first shot and completion of HPV vaccine within six months of receiving the interventions. Repeated measure ANOVA was utilized to examine the intervention effect on knowledge between intervention and control groups. Knowledge differed significantly, with participants in the intervention group demonstrating the greatest improvement following the intervention. A multivariable logistic regression was used to examine the association between HPV vaccine initiation and study group assignment. There was a significant effect of provider recommendation, parent’s gender, and health insurance status on HPV vaccine uptake. This study demonstrated positive impact of a culturally tailored intervention on HPV vaccination uptake among Chinese Americans.

## Introduction

In the United States, human papillomavirus (HPV) is the most common sexually transmitted infection (STI), affecting an estimated 79 million Americans [1]. Each year there are an estimated 14 million new cases of HPV in the United States [2]. HPV is most prevalent among teenagers and young adults [1]. It is associated with genital warts and various forms of cancer, such as cervical, penile, and oropharyngeal cancer [3]. There are more than 40 strains of HPV with varying implications on human health. High-risk strains, HPV-16 and HPV-18, are strongly associated with cancer development. The estimated incidence of cancers related to HPV is 19,400 for women and 12,100 for men [1, 3, 4]. Among these malignancies, cervical cancer (CC) is the most common. For CC specifically, the incidence rate in the United States is 8 cases per 100,000 persons [5].

Improving vaccination rates could eradicate CC by offering improved protection against the most harmful strains of HPV [2]. A report in England found that young female adults between ages 19 and 21 and ages 22 and 24 experienced tremendous declines in the prevalence of HPV-16 and -18 following the 10-year mark of the HPV vaccine adoption [6]. To prevent the onset of CC and HPV, the Advisory committee on immunization practices (ACIP) established recommendations for adolescent uptake of the HPV vaccine and now recommends vaccine initiation between ages 11 and 12; the vaccine can be initiated as young as age 9. In addition, the HPV vaccine is recommended for women between ages 13 and 26 and for men between ages 13 and 21 [2, 7].

HPV vaccination rates for Asian American adolescents remain suboptimal despite increasing public health efforts in the past decade. The estimated completion rate for two or three doses of HPV vaccine was 60.1% for adolescent Asian American females between ages 13 and 15 [8]. The completion rates for Asian American adolescent males was 41% [9]. Completion rates for both male and female Asian American adolescents suggest significant room for improvement to reach the goal of 80% vaccination rates established in the Healthy People 2020 plan [8, 9]. A previous study conducted in California found low vaccine awareness and low vaccine initiation and completion among Chinese Americans [10]. In addition, several studies have found lower rates of HPV vaccine uptake among Asian Americans when compared to other racial groups in the United States [11, 12]. Given the low rates of HPV vaccination among Asian Americans, it is imperative to conduct studies in this population with hopes of bridging gaps in vaccine utilization to optimize prevention of the harmful complications associated with HPV.

Given that the recommendations established by ACIP apply to minors (persons under age 18), it is critical to engage parents to promote HPV vaccination awareness and uptake. Previous studies have identified factors associated with parents’ intention toward and update of HPV vaccine, including parent gender, income, education, and HPV-related knowledge and children’s health insurance coverage. For example, previous studies have identified lack of HPV-related knowledge as a factor inhibiting parents from making informed decisions on HPV vaccination for their children [10, 11, 13–16]. Researchers also found that higher family income [15] and higher parental education [15, 17] are associated with higher HPV-related knowledge and vaccine initiation and completion rates. In addition, health insurance coverage for children is related to HPV vaccination uptake.

An external factor that plays a critical role in parents’ decision-making about HPV vaccination is health providers’ recommendation [15, 17]. HPV vaccination recommendation has been consistently associated with greater parental intention of and actual HPV vaccine uptake in various groups [18–20], including Asian Americans [21, 22].

Lastly, among immigrant populations, acculturation may play a role in parents’ intention of and actual uptake of HPV vaccine. Previous research has shown that acculturation factors, such as country of birth, years living in the United States, English proficiency, and cultural beliefs affect individual knowledge, awareness, and acceptability of the HPV vaccine [23–26].

Guided by community-based participatory research (CBPR), we designed and implemented an intervention to raise awareness of HPV vaccine among Chinese American parents or guardians and to promote uptake of HPV vaccination among their children. The objective of this study was to evaluate the effect of the intervention among Chinese Americans, including its efficacy among Chinese American parents/guardians.

## Methods

### Study Design

We designed and conducted a longitudinal pilot study to evaluate the efficacy of an HPV vaccination intervention among Chinese American parents and guardians of teenagers. Specifically, we used multilevel and trauma-informed approaches to engaging parents/guardians, healthcare providers, adolescents, and community leaders in the planning, development, and implementation process. We recruited Chinese American community members who self-identified as a Chinese parent or guardian and who had at least one child between ages 11 and 18 who had ever received HPV vaccine. Guided by community-based participatory research (CBPR) principles, we collaborated with a community health centers that predominantly serves low-income Chinese American community in Philadelphia. In collaboration with health providers at the community health center, we designed the program with age, culturally and linguistically appropriate content and delivery modalities. A total of 180 participants were recruited for this study with 110 in the intervention group and 70 in the control group. The intervention group received an HPV specific intervention, while the control group received a general health intervention.

### Multilevel and Multimedia Intervention

Working closely with the parents/guardians, healthcare providers, adolescents, and community leaders, we designed a culturally tailored behavioral intervention, which included four major components.

#### Interventional Videos

Two multimedia educational videos were developed and tailored to reflect Chinese social and cultural contexts. From watching the videos, we reinforced parents’ awareness, knowledge, attitude, intention, and actual uptake of HPV vaccination. The first video included interactive educational modules on HPV vaccination, formatted with tailored messages and dialogue among pediatricians, parents, and female/male adolescents.

- Module 1, pediatrician to parents. In this module, a well-known pediatrician demonstrated the routine physical exam and vaccine injection for a child.
- Module 2, conversation about the HPV vaccine. This conversation happened between two parents. Two moms talked about the HPV vaccine, their confusions and concerns about the HPV vaccine, the knowledge that they gained from the doctor, and their decisions to take the HPV vaccine for their children.
- Module 3, communication in parents to adolescents. This module was to activate parents’ and children’s motivation about taking the HPV vaccine by discussing the benefits of taking the vaccine.
- Module 4, conversation between two adolescents. A perspective from kids about their interpretation about the HPV vaccine, what they care about most concerning the vaccine, and the factors that affect their intention to take the HPV vaccine.

The second video was an interview of a well-known pediatrician. The discussion encompassed several topics that most concerned Chinese parents, including: (1) what do most parents want to know about the HPV vaccine; (2) safety of the HPV vaccine; (3) HPV vaccine and sexual activity; (d) what do children worry about; (4) why children need three shots; (5) why children need to receive the vaccine earlier; and (6) what is the most crucial point for parents to know about the vaccine.

#### Interactive Discussion

After participants watched two videos, they typically would have many questions that they wanted to discuss with the health educator. The interactive discussion enabled participants to reflect on what they learned and reinforce the knowledge that they learned from the two videos.

#### Bilingual HPV Printed Educational Materials

Besides the interventional videos, four printed materials were adapted from the Center for disease control and prevention (CDC). Participants could share these with their family members and friends to learn more about HPV vaccination. The printed materials included: (1) HPV vaccine is for girls and boys; (2) what HPV can cause; (3) the primary data of HPV infection, and (4) vaccine tracking chart for parents’ use. The health educator distributed these materials at the end of each interventional session.

#### Text Message Reminders to Participants

After the initial intervention session, we also sent four reminder text messages, in both English and Chinese, to participants that included more information on HPV vaccine benefits and HPV vaccine access.

### Data Collection

We administered three assessments to study participants at baseline (pre-intervention), post-intervention, and six-month follow-up. We administered the baseline and post-intervention surveys to participants at the community health centers. Each assessment was approximately 20 min in length, and participants had the option of answering the questions in either English or Chinese. Onsite language assistance was available from a trained bilingual community health educator. We administered a six-month follow-up assessment to study participants by telephone. Informed consent forms and HIPAA authorization forms were obtained from each eligible participant who volunteered to participate in the study. This study was reviewed and approved by Temple University Institutional Review Board (protocol number PT 22842).

### Measurement

#### Outcome Measures

The primary outcome of interest for this study was HPV vaccine uptake assessed at six-month follow-up. Specifically, at the six-month follow-up assessment, we measured whether any children aged 11 to 18 of the participants had initiated the HPV vaccine schedule. Additionally, we measured whether any of the children had completed the HPV vaccine schedule, i.e., received all 3 shots. Both outcome measures are dichotomous variable (yes v. no).

#### Baseline Measures

We collected information on study participants’ socio-demographics, such as age in years, educational attainment (whether they had a high school degree), annual household income (less than $20,000 v. greater than $20,000), years living in the United States (binary; less than 15 years v. greater than 15 years), health insurance status (yes v. no), and sex (male v. female). In addition, we used seven questions to assess participants’ knowledge of HPV and HPV vaccine. We summed their answers to compute an HPV knowledge score, which ranged from 0 to 7, with a higher numeric value indicating a higher level of knowledge. We also asked participants whether their children’s pediatrician had recommended HPV vaccine (yes v. no). In addition, we assessed participants’ intention toward HPV vaccine uptake for their children (yes v. no).

#### Post-Intervention Measures

Following the completion of the interventions, we administered a post-intervention survey covering the same questions on HPV-related knowledge.

### Statistical Plan

We used t-tests and chi-square tests to compare the sociodemographic and health-related characteristics of participants between the intervention and control group. We conducted repeated measure analysis of variance (ANOVA) to examine the intervention effects on HPV-related knowledge. Finally, we used binary logistic regression to examine the intervention effects on the initiative of HPV vaccine schedule at six-month follow-up. All analyses were conducted in Stata 16 [27], except for the repeated measure ANOVA, which was conducted in SPSS 24 [28].

## Results

As results in Table 1 show, there were no significant differences between intervention and control groups for all sociodemographic factors except for age. The control group were on average 3.29 years older than the intervention group. Overall, the majority of the study participants were female, had lived in the United States for more than 15 years, did not have a high school degree, and had health insurance coverage. Only about one-third of the parents or guardians reported that they received HPV vaccine recommendations from their children’s pediatrician.

**Table 1:** Sociodemographic and health-related characteristics of participants, by study group (N = 180)

	Intervention n (%) or mean (sd)	Control n (%) or mean (sd)	*t* (*df*), *p* $${\chi }^{2}$$ (*df*), *p*
Age	42.09 (0.73)	45.38 (1.21)	2.49 (175), .02
Sex			1.72 (1), .19
Male	35 (31.8%)	29 (41.4%)	
Female	75 (68.2%)	41 (58.6%)	
Years in the U.S.			.75 (1), .39
≤15 yrs	55 (50.0%)	29 (43.3%)	
> 15 yrs	55 (50.0%)	38 (56.7%	
Education			.12 (1), .73
Below high school	61 (55.45%)	37 (52.9%)	
High school or above	49 (44.55%)	33 (47.1%)	
Income			2.05 (1), .15
0–$19,999	56 (50.91%)	28 (40.00%)	
$20,000 or more	54 (49.09%)	42 (60.00%)	
Health insurance			2.11 (1), .15
Yes	101 (91.82%)	68 (97.14%)	
No	9 (8.18%)	2 (2.86%)	
Pediatrician recommended HPV vaccine			.17 (1), .68
Yes	36 (32.7%)	25 (35.7%)	
No	74 (67.27%)	45 (64.29%)	

*sd* standard deviation, *df* degree of freedom

We also examined the changes in mean HPV knowledge scores from baseline to post-intervention, by study group. Results in Table 2 show that HPV knowledge scores increased for both groups (p < 0.001), but the magnitude was significantly higher for the intervention group, with an increase of 4.29, compared to an increase of 0.73 for the control group (p = 0.001). This indicates a significant intervention effect on the HPV-related knowledge.

**Table 2:** Changes in HPV-related knowledge, by study group (N = 180)

	Intervention			Control			Repeated measure ANOVA
	Baseline	Post	t (df), p	Baseline	Post	t (df), p	F (df), p
HPV knowledge score	1.26 (1.74)	5.55 (2.10)	− 17.49 (109), < .001	0.96 (1.54)	1.69 (2.16)	− 3.33 (69), < .001	101.19 (1), .001

*df* degree of freedom, *ANOVA* analysis of variance

At six-month follow-up, we assessed if any adolescent children of the participants initiated or completed the HPV vaccine. Results presented in Table 3 show that 72 participants (65.45%) in the intervention group reported having at least one child aged between 11 and 18 that initiated and completed the vaccine schedule. In comparison, only two participants (2.9%) in the control group reported initiation of the vaccine schedule for their children, and none completed the schedule. Chi-square test results showed that the initiation rate was significantly higher in the intervention group than that in the control group (p < 0.001).

**Table 3:** HPV vaccine uptake rates at 6-month follow-up, by study group (N = 180)

	Intervention n (%)	Control n (%)	${\chi }^{2}$(*df*), *p*
Any children aged 11–18 *initiated* vaccine schedule			69.24 (1) < .001
Yes	72 (65.45%)	2 (2.9%)	
No	38 (34.55%)	68 (97.1%)	
Any children aged 11–18 *completed* vaccine schedule			n/a
Yes	72 (65.45%)	0 (0.0%)	
No	38 (34.55%)	70 (100.0%)	

*df* degree of freedom

Logistic regression results presented in Table 4 show significant intervention effects on the initiation of the HPV vaccine schedule for at least one child. Specifically, the odds of children’s HPV vaccine initiation for participants in the intervention group was 485.44 times of the odds for those in the control group, with other variables held constant. In addition, three covariates in the regression model were significantly associated with vaccine initiation. Pediatrician’s recommendation was associated with a higher likelihood of reporting vaccine initiation at follow-up (odds ratio: 3.45, 95% CI: 1.03 – 11.53). Interestingly, female parents or guardians were only about one-third (odds ratio: 0.32, 95% CI: 0.11 – 0.90) as likely as male parents or guardians to report vaccine initiation. Health insurance coverage was positively associated with vaccine initiation (odds ratio: 11.05, 95% CI: 2.38 – 108.16).

**Table 4:** Logistic regression on the probability of any children aged 11 to 18 initiating the HPV vaccine schedule

Predictors	Odds ratio (95% CI)
Intervention group (ref: control)	485.44 (43.26, 5,447.54)***
Pediatrician’s recommendation (ref: no)	3.45 (1.03, 11.53)*
HPV knowledge score	0.75 (0.56, 1.02)
Intention for HPV vaccine (ref: no)	0.26 (0.04, 1.80)
Age (in years)	0.98 (0.92, 1.03)
Female (ref: male)	0.32 (0.11, 0.90)*
High school or above (ref: less than high school)	0.44 (0.16, 1.17)
Annual household income≥$20 k (ref: < $20 k)	0.56 (0.21, 1.50)
Years lived in U.S. > 15 years (ref: < 15 years)	1.74 (0.68, 4.44)
Health insurance (ref: no)	16.05 (2.38, 108.16)**
Intercept	0.02
Likelihood ratio statistics (df), p	105.43 (10), < .001
Pseudo R^2^	0.45

*CI* confidence interval, *ref* reference group, *df* degree of freedom

*p < .05; **p < .01; ***p < .001

## Discussion

This study evaluated the effects of intervention among Chinese American parents or guardians to children between the age of 11 and 18, to promote HPV vaccine uptake among Chinese American teenagers. We found that the culturally appropriate intervention had significant effects on HPV-related knowledge among parents or guardians, and HPV vaccine uptake among their children. This is consistent with the increasing body of literature on the efficacy of community-based, culturally appropriate interventions in promoting HPV vaccination among the underprivileged, underserved populations [29–32]. It is noteworthy that our intervention achieved a 65% initiation and completion rate, which was much higher than initiation and completion rates in a Chinese American sample (25% initiation rate and 9% completion rate) from a previous study [10].

In addition, our study findings attest to the effectiveness of an HPV-focused intervention when compared to general health education. The HPV-related knowledge of parents or guardians was significantly associated HPV vaccine initiation among children (results not shown). The significance of this association, however, was lost when we added study group assignment into the model. This suggests that increased HPV-related knowledge of parents or guardians directly leads to HPV vaccine uptake. However, it is also possible that knowledge directly affected vaccine uptake through other psychological factors, such as self-efficacy and perceived risks/susceptibility [33, 34]. Previous research has suggested complicated mechanisms through which various psychosocial factors interact to affect health behaviors such as screening and vaccination [35–39].

Health insurance status was the second strongest predictor for HPV vaccine initiation in our study. This finding was consistent with the results of a systematic review, which identified health insurance status as a predictor for HPV vaccine initiation and completion [40]. We also found that pediatrician’s recommendation for HPV vaccine was a significant predictor of HPV vaccine initiation, which was also consistent with previous findings [19, 20]. The role of health insurance and healthcare provider recommendations echoes the significance of having the capacity to access healthcare services as well as the ability to afford coverage that accommodates individual need. These findings shed light on future interventions that aim to address barriers to HPV vaccination on multiple levels, from health system to health provider to the individual level. Specifically, policy makers and public health efforts need to address systematic barriers to health insurance, especially among underprivileged and underserved communities; provide better access to health care resources that are culturally and linguistically appropriate in immigrant communities; and enhance health provider awareness and knowledge of HPV. To meet the Healthy People 2020 goal and increase HPV vaccination rates nationwide, multi-level intervention efforts are urgently needed.

Moreover, parent gender was a significant predictor of HPV vaccine uptake. Female parents were less likely to report vaccine uptake compared to male parents or guardians. This finding contradicts the existing literature, as a previous study found that male parents were less likely than female parents to report vaccine uptake among adolescents [20]. One possible explanation is that male parents or guardians played a more effective role in the decision-making process of HPV vaccine uptake in the Chinese American community. More research is needed to test this theory.

This study is not without limitations. This study formed a community sample through convenient sampling. Therefore, the findings could not be generalized to the greater population. We recruited the participants at a community-based federally qualified health center, which might lead to sampling bias. Specifically, we might have under-sampled those who were less medically aware and those who utilize healthcare services through other health providers. Future iterations of this study could benefit from planning for a more expansive repertoire of community-based organizations and healthcare entities to capture a wider range of individuals from the target population.

## Conclusions

In conclusion, we found that our culturally tailored intervention significantly increased HPV-related knowledge among Chinese American parents or guardians and increased HPV vaccine uptake among their adolescent children. The findings of this study contribute to the growing body of the literature on the efficacy of culturally tailored interventions in promoting cancer preventive behaviors in medically underserved populations.
